# High mutation burden of circulating cell‐free DNA in early‐stage breast cancer patients is associated with a poor relapse‐free survival

**DOI:** 10.1002/cam4.3258

**Published:** 2020-06-29

**Authors:** Jouni Kujala, Jaana M. Hartikainen, Maria Tengström, Reijo Sironen, Veli‐Matti Kosma, Arto Mannermaa

**Affiliations:** ^1^ Institute of Clinical Medicine, Pathology and Forensic Medicine University of Eastern Finland Kuopio Finland; ^2^ Translational Cancer Research Area University of Eastern Finland Kuopio Finland; ^3^ Institute of Clinical Medicine, Oncology University of Eastern Finland Kuopio Finland; ^4^ Cancer Center Kuopio University Hospital Kuopio Finland; ^5^ Department of Clinical Pathology Kuopio University Hospital Kuopio Finland; ^6^ Biobank of Eastern Finland Kuopio University Hospital Kuopio Finland

**Keywords:** biomarker, liquid biopsy, prognosis, recurrence, serum

## Abstract

**Background:**

High tumor mutation burden is shown to be associated with a poor clinical outcome. As the tumor‐derived fraction of circulating cell‐free DNA (cfDNA) is shown to reflect the genetic spectrum of the tumor, we examined whether the mutation burden of cfDNA could be used to predict the clinical outcomes of early‐stage breast cancer (BC) patients.

**Methods:**

We selected a set of 79 Finnish early‐stage BC cases with a good prognosis based on traditional prognostic parameters but some of which still developed relapsed disease during follow‐up. cfDNA was isolated from the serum collected at the time of diagnosis, sequenced, and compared to matched primary tumors, clinical parameters, and survival data.

**Results:**

High cfDNA mutation burden was associated with the poor relapse‐free survival (RFS) (*P* = .016, HR = 2.23, 95% Cl 1.16‐4.27) when patients were divided into high and low mutation burden according to the median number of somatic variants. A high discordance was observed between the matched tumor and cfDNA samples, thus highlighting the challenges related to the liquid biopsy of early‐stage cancer cases. Despite the low number of detected tumor‐specific variants, the presence of tumor‐specific somatic variants in the cfDNA was associated with the poor RFS (*P* = .009, HR = 2.31, 95% Cl 1.23‐4.31).

**Conclusions:**

Our results confirm previously observed challenges about the accuracy of liquid biopsy‐based genotyping of early‐stage cancers and support the parallel sequencing of tumor and cfDNA while also demonstrating how the presence of tumor‐specific somatic variants and the high mutation burden in the cfDNA are both associated with the poor RFS, thus indicating the prognostic potential of liquid biopsy in the context of early‐stage cancers.

## Graphical abstract

High mutation burden and the presence of tumor‐specific mutations in circulating cell‐free DNA in early‐stage breast cancer patients were observed to be associated with a poor relapse‐free survival. Our results suggest that the liquid biopsy of early‐stage cancers might provide potential benefits when making primary prognosis despite the challenges that are associated with the liquid biopsy of early‐stage cancers.

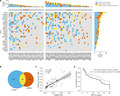

## 1 INTRODUCTION

Breast cancer (BC) is a heterogeneous disease with a high degree of phenotypic variation within individual primary tumors.^1^ This diversity, often referred to as intratumoral heterogeneity (ITH), arises largely from somatic driver variants that provide a selective advantage for variant‐carrying cancer cell in its microenvironment.^2^ Fitness‐promoting variants may lead to positive selection and clonal expansion of cancer cell lineages as described by previous studies of cancer genomics.^3^, ^4^, ^5^ As a result, individual BC tumors tend to be composed of multiple subpopulations that may respond differently to treatments and thus pose a major challenge for targeted cancer therapies where treatment strategies are selected based on specific biomarkers.^6^

It has been largely hypothesized that intensive ITH could reflect cancer's potential for evolutionary adaptation and thus be associated with a poor clinical outcome. Indeed, recently The Cancer Genome Atlas Network (TCGA) data utilizing pan‐cancer studies^7^, ^8^ have reported that high ITH is associated with a poor patient survival in various cancer types, thus highlighting the potential prognostic importance of ITH.

The current approaches for ITH testing strongly rely on tumor samples obtained from needle biopsies and surgical excisions. Tumor biopsies are known to have their limitations and the analysis of circulating cell‐free DNA (cfDNA) is often proposed as a minimally invasive and easily repeatable alternative for tumor biopsies.^9^ cfDNA is released into circulation from the apoptotic and necrotic tumor cells and a small fraction of it is demonstrated to carry tumor‐specific genetic alterations that can be detected already in an early‐stage disease.^10^, ^11^

We hypothesize that the mutational spectrum of cfDNA reflects the mutational spectrum of tumor and predicts the clinical outcome of early‐stage BC patient in a manner similar to primary tumors. To test our hypothesis, we sequenced a set of primary tumors and peripheral cfDNA samples of early‐stage BC patients with a stage T1N0M0 or T2N0M0 disease and compared results to clinical data. Our results indicate that the cfDNA mutation burden and the presence of tumor‐specific somatic variants in cfDNA are both associated with a poor relapse‐free survival (RFS) thus providing further evidence that liquid biopsy could be used to identify early‐stage patients with a higher risk of relapse and to assess more precise prognosis.

## 2 MATERIALS AND METHODS

### 2.1 Patients, sample material, and clinical data

This study included a set of 79 Eastern Finnish breast cancer patients who had no nodal or distant metastases at the time of primary diagnosis with a stage T1N0M0 or T2N0M0. Clinical data, tissue, blood, and serum samples were obtained from the Kuopio Breast Cancer Project (KBCP), a prospective population‐based case‐control study conducted in 1990‐1995 in Eastern Finland.^12^, ^13^, ^14^ Patient demographics are presented in Table 1. This research project was advocated by the Research Ethics Committee of the University of Eastern Finland and Kuopio University Hospital. All participants who have participated in project have given their knowledge‐based written consent to participation.

**TABLE 1:** Patient demographics

Characteristic	Relapsed cases N (%)	Non‐relapsed cases n (%)
Number of cases	45 (100.0)	34 (100.0)
Age at diagnosis		
≤39	5 (11.1)	2 (5.9)
40‐49	14 (31.1)	8 (23.5)
50‐59	15 (33.3)	9 (26.5)
60‐69	6 (13.3)	8 (23.5)
≥70	5 (11.1)	7 (20.6)
Estrogen receptor (ER) status		
Negative	5 (11.1)	5 (14.7)
Positive	40 (88.9)	29 (85.3)
Progesterone receptor (PR) status		
Negative	12 (26.7)	11 (32.4)
Positive	33 (73.3)	23 (67.6)
HER2—receptor status		
Negative	38 (84.4)	29 (85.3)
Positive	3 (6.7)	2 (5.9)
Missing	4 (8.9)	3 (8.8)
Triple‐negative cases		
Yes	2 (4.4)	3 (8.8)
No	41 (91.1)	28 (82.4)
Missing	2 (4.4)	3 (8.8)
Tumor grade		
I	11 (24.4)	6 (17.6)
II	28 (62.2)	20 (58.8)
III	6 (13.3)	8 (23.5)
Stage		
I	35 (77.8)	25 (73.5)
II	10 (22.2)	9 (26.5)
Tumor size		
T1	35 (77.8)	25 (73.5)
T2	10 (22.2)	9 (26.5)
Nodal status		
N0	45 (100.0)	34 (100.0)
Distant metastasis		
M0	45 (100.0)	34 (100.0)
Histological type		
Ductal	38 (84.4)	32 (94.1)
Lobular	3 (6.7)	1 (2.9)
Tubular	2 (4.4)	1 (2.9)
Mixed (ductal and lobular)	2 (4.4)	0 (0.0)
Radiotherapy^a^		
Yes	19 (42.2)	9 (26.5)
No	26 (57.8)	25 (73.5)
Chemotherapy^a^		
Yes	0 (0.0)	0 (0.0)
No	45 (100.0)	34 (100.0)
Hormonal therapy^a^		
Yes	0 (0.0)	0 (0.0)
No	45 (100.0)	34 (100.0)

^a^ Refers to treatment received after tumor and serum sampling. Patients did not receive any treatment prior to sampling.

### 2.2 DNA extraction

cfDNA was extracted from 79 patient serum samples using the QIAamp Circulating Nucleic Acid kit (Cat No. 55114, Qiagen). Genomic DNA (gDNA) from 61 formalin‐fixed and paraffin embedded (FFPE) tumor sections was extracted using High Pure FFPET DNA Isolation Kit (Cat No. 06650767001, Roche Diagnostics, Indianapolis, IN, USA) and gDNA from 10 blood samples was extracted using QIAamp DNA Blood Midi Kit (Cat No. 51185, Qiagen). All DNA extractions were made according to the manufacturers’ protocols. Quality of extracted samples was assessed with Nanodrop ND‐1000 (Thermo Fischer Scientific) and Qubit 2.0 (Invitrogen). cfDNA samples were further analyzed with TapeStation 4200 electrophoresis system with a D5000 High Sensitivity ScreenTape Assay (Cat No. 5067‐5592 and 5067‐5593, Agilent Technologies) to identify possible gDNA contaminations.

### 2.3 Library preparation and sequencing

Sequencing libraries from cfDNA samples were prepared using QIAseq cfDNA Library Kit (Cat No. 180015, Qiagen) according to the manufacturer's protocol. Libraries with unique indices were pooled with xGen Universal blocking oligos (Cat No. 1075474, Integrated DNA Technologies) and custom SureSelectXT2 target capture baits (Cat No. 5190‐4807, Agilent Technologies) targeting 106 genes associated with metastatic BC (File S1). Hybrid capture reaction was performed with SureSelectXT2 Target Enrichment System Kit (Cat No. G9621B, Agilent Technologies) according to the manufacturer's protocol. Captured cfDNA libraries were on‐beads amplified with GeneRead DNA I Amp Kit (Cat No. 180455, Qiagen) and further purified with Agencourt AMPure XP beads (Cat No. A63881, Beckman Coulter). Sequencing libraries from the BC tumor and blood samples were prepared and enriched using HaloPlex Target Enrichment System (Cat No. G9961C, Agilent Technologies) with custom amplicons targeting 25 BC associated consensus genes (File S2) according to the manufacturer's protocol. Agilent NGS FFPE QC Kit (Cat No. G9700B, Agilent Technologies) was used for determining the DNA integrity scores and the amount of input FFPE‐derived gDNA for the library preparation, according to the manufacturer's protocol. All libraries were quantified using Bioanalyzer High sensitivity DNA Kit (Cat No. 5067‐4626, Agilent Technologies) and sequenced with Illumina NextSeq and MiSeq sequencing platforms (Illumina) located at the Genome Center of Eastern Finland, University of Eastern Finland, Finland.

### 2.4 Somatic variant calling

Paired‐end reads were trimmed with cutadapt^15^ and mapped to hg19 reference genome with BWA‐MEM.^16^ Mapped reads with a Phred quality score < 20 were filtered and remaining reads were sorted and indexed with SAMtools.^17^ Local realignment was performed with GATK IndelRealigner^18^ tool to minimize the number of mismatching bases across all reads. FASTQ and BAM file qualities were assessed with FastQC and Picard CollectHsMetrics. Variant calling was performed using VarScan2^19^; at least 10 variant supporting reads and variant allele frequency of 0.01 were required to retain variant. Variants reported in Finnish population in ExAC^20^ or detected in sequenced blood samples were filtered to obtain somatic variants calls. Called somatic variants were annotated with ANNOVAR^21^ and public databases. Pathogenicity of somatic variants was evaluated with existing ClinVar,^22^ COSMIC^23^ and International Cancer Genome Consortium (ICGC)^24^ records. Pathogenicity of somatic variants without existing database records was evaluated with MetaSVM^25^ scoring where the score of 0‐0.825 was interpreted as likely pathogenic variant and score greater 0.825 was interpreted as pathogenic variant. The computational analyses were run on the servers provided by the Bioinformatics Center, University of Eastern Finland, Finland.

### 2.5 Statistical analysis

The overall mutation burden of samples was estimated by calculating the number of somatic variants per number of sequenced base pairs. Somatic variants detected both in the matched tumor and cfDNA were referred as tumor‐specific somatic variant. The linear correlation between two variables was measured with Pearson's correlation coefficient (PCC). The differences in group means were compared with an unpaired *t* test. Diagnostic ability of binary classifiers was estimated with receiver operating characteristic (ROC) curve. Survival data were analyzed using the multivariate Cox regression analysis in IBM SPSS Statistics 25 (IBM). All multivariate analyses were stratified with age at the time of diagnosis, grade, stage, estrogen receptor (ER) status, progesterone receptor (PR) status, human epidermal growth factor receptor 2 (HER2) status and radiotherapy. The overall survival (OS) and breast cancer‐specific survival (BCSS) were calculated as the time from the date of diagnosis to the date of last follow‐up or death; cause of death was coded either caused by BC or not caused by BC. Relapse‐free survival (RFS) was calculated as the time of diagnosis to the time of first local or distant relapse or new BC. A *p*‐value of .05 (two‐sided) or less was considered statistically significant. Numerical values are presented as mean ± standard error of the mean (SEM).

## 3 RESULTS

### 3.1 Patient cohort

All included patients were female with a median age of 55 years. Most of the tumor samples had positive ER status (87.3%), positive PR status (70.9%), and negative HER2 status (84.8% of cases with available information). In overall, five cases (6.3%) had been diagnosed with a triple‐negative breast cancer. All patients had been diagnosed either with stage I (75.9%) or stage II (24.1%) disease. The following histological subtypes were involved in the study: 70 ductal carcinomas (88.6%), four lobular carcinomas (5.1%), three tubular carcinomas (3.8%) and two mixed ductal and lobular carcinomas (2.5%). All patients had undergone the surgical removal of the tumor and 28 patients (35.4%) had received radiotherapy after surgery. None of the patients had received chemotherapy or hormonal therapy before or after surgery. Median RFS and OS within cohort were 14.1 and 18.3 years.

### 3.2 Sequencing performance

The ratios of targeted bases covered with more than 100 reads after read processing were 94.6 ± 0.4%, 93.3 ± 0.3%, and 80.7 ± 0.4% for cfDNA, blood, and tumor, respectively. The achieved mean sequencing coverages after read processing were 3557 ± 69x for cfDNA samples, 887 ± 38x for tumor samples and 1669 ± 179x for blood samples (File S3).

### 3.3 Detected somatic variants

Somatic single nucleotide variants or indels were detected in 93.4% (57 out of 61) of the primary tumors and in 83.5% (66 out of 79) of cfDNA samples. On average, we detected 5.1 ± 0.4 somatic variants per tumor sample (25.4 ± 2.0 mut/Mbp) and 4.2 ± 0.4 somatic variants per cfDNA sample (9.4 ± 0.9 mut/Mbp). The frequency of detected somatic variants was significantly (*P* = .004, unpaired *t* test) higher in relapsed cfDNA samples when compared to patients without relapse. Similar difference was not observed between relapsed and non‐relapsed tumor samples (*P* = .355, unpaired *t* test) despite the statistically significant correlation (*r* = .274, *P* = .032) observed between the mutation burdens of matched tumor and cfDNA samples.

**FIGURE 1:**
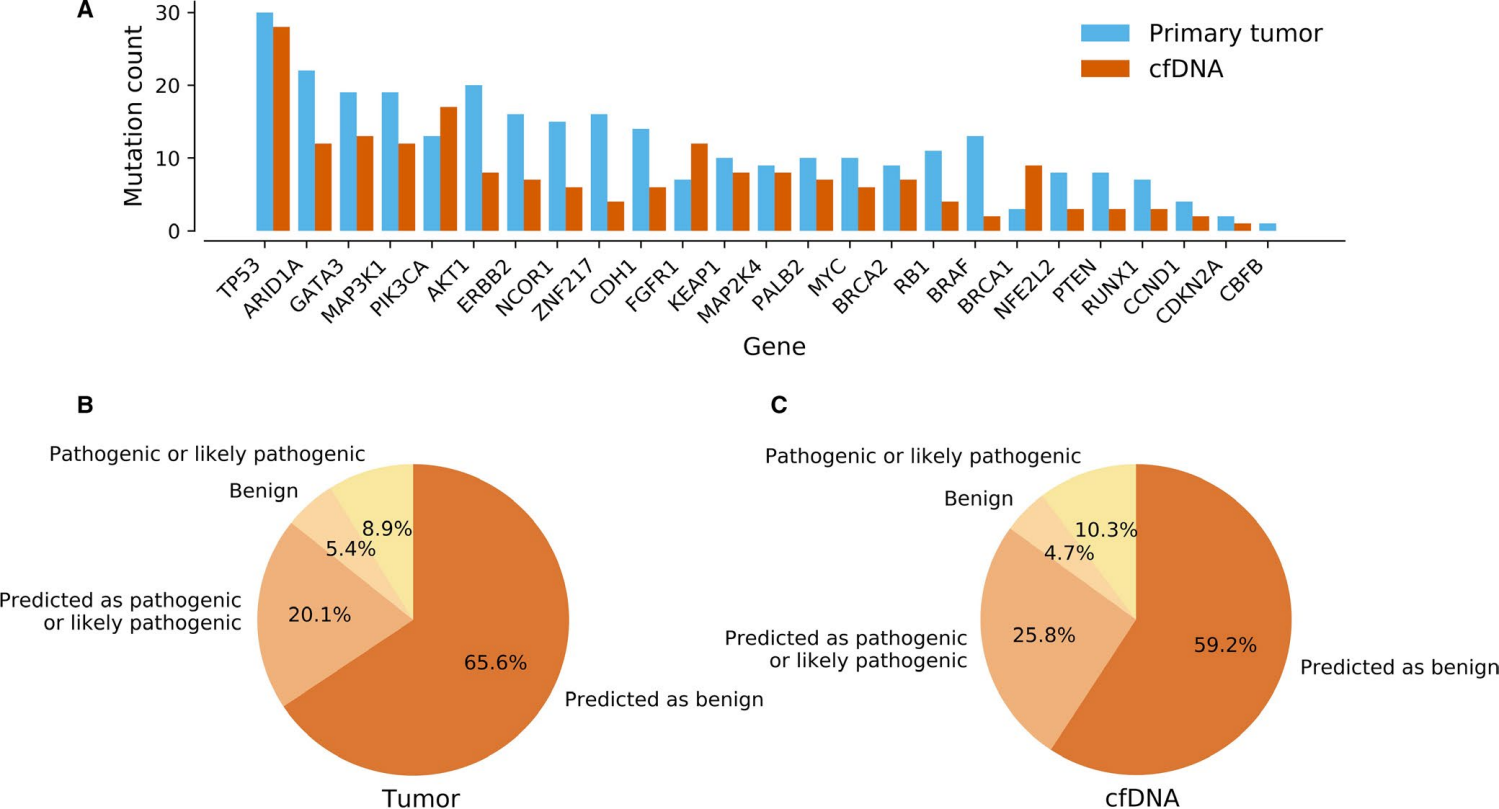
Distribution of somatic variants per gene. *TP53* was the most commonly mutated gene both in the tumor and cfDNA samples while the mutation frequency of other genes varied slightly between samples (A). Only genes sequenced in both samples are shown in figure. Only about 15% of somatic variants detected in the tumor (B) or cfDNA (C) had an existing clinical record in public databases while variants without public records were annotated as variant of uncertain significance (VUS). According to our prediction, about 60% of all VUSs were predicted to be benign in their nature while the rest of the VUSs possessed potential pathogenic potential

An average variant allele frequency (VAF) of somatic variants ranged between 1.1%‐73.8% in tumor and 1.0%‐4.2% in cfDNA. The average VAF was 9.3 ± 0.8% in tumor and 1.7 ± 0.1% in cfDNA when all sequenced genes and detected somatic variants were considered. Most frequently mutated genes were *TP53* (10.1% of all variants), *ARID1A* (7.4%), *AKT1* (6.8%), *GATA3* (6.4%), and *MAP3K1* (6.4%) in tumor (Figure 1A, Figure S4) and *TP53* (8.6%), *PIK3CA* (5.2%), *GATA3* (4.0%), *ARID1A* (3.7%), *FGFR1* (3.7%), and *MAP3K1* (3.7%) in cfDNA (Figure S5). In total, 8.9% of all somatic variants detected in the tumor and 10.3% of all somatic variants detected in the cfDNA were annotated as likely pathogenic or pathogenic based on their existing records in public databases (Figure 1A,B). The ratios of known benign somatic variants in tumor and cfDNA were 4.7% in tumor and 5.4% in cfDNA correspondingly. Remaining variants (85.7% in tumor and 85.0% in cfDNA samples) did not have any existing records in public databases and were considered as variants of uncertain significance (VUS) whose pathogenicity was predicted computationally. According to prediction, 23.4% of the VUS detected in the tumor and 30.3% of VUS detected in the cfDNA were annotated as likely pathogenic or pathogenic while rest of the variants were annotated as benign.

### 3.4 High mutation burden of tumor is associated with the poor RFS and BCSS

**FIGURE 2:**
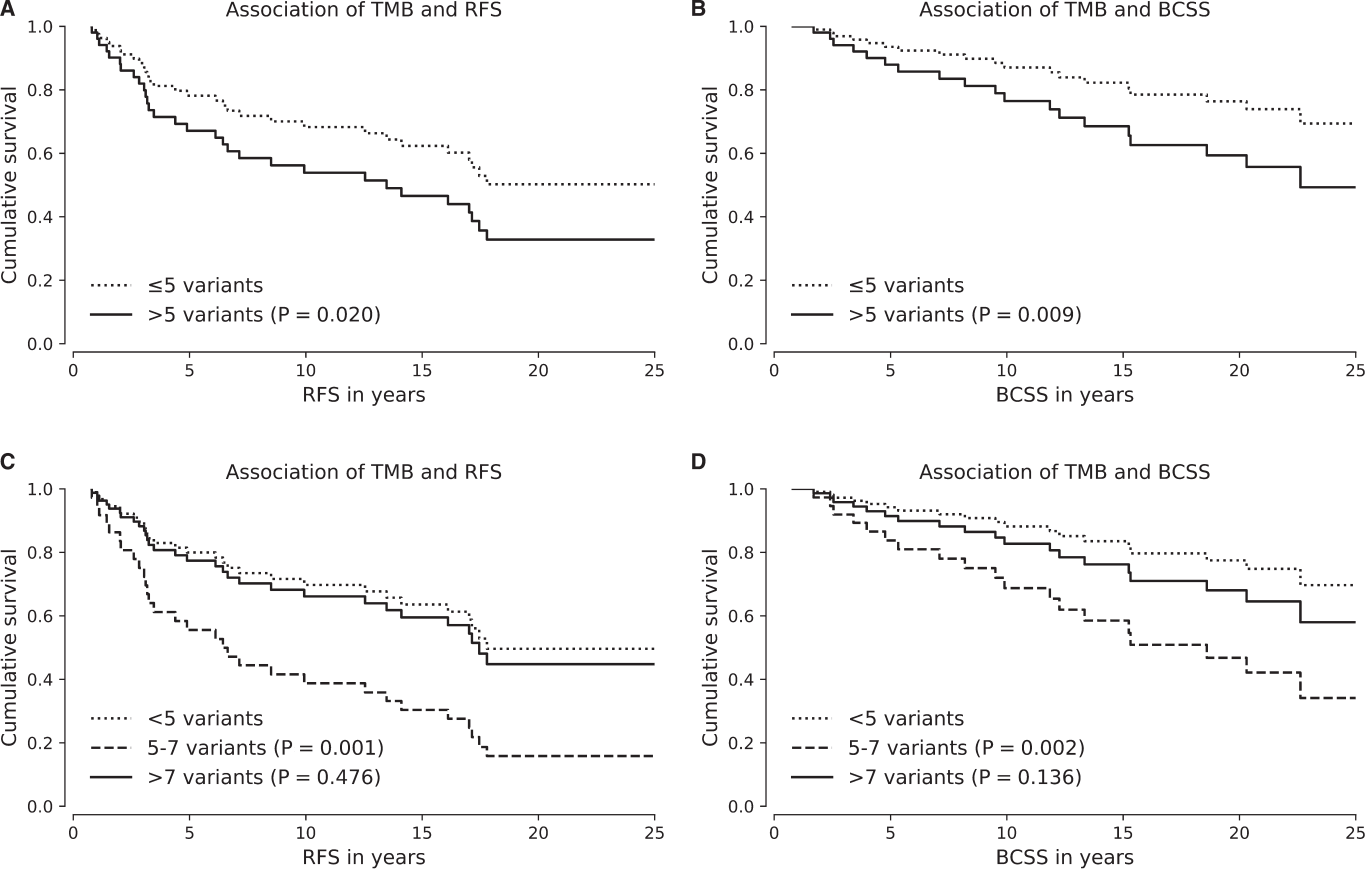
Association of tumor mutation burden with RFS and BCSS. When patients were divided into two groups, high and low, according to the median tumor mutation burden, high tumor mutation burden (>5 variants) was associated with a poor RFS (A) and BCSS (B). Further analysis observed that the association of highest two quartiles and survival was inconsistent with the hypothesis as the intermediate tumor mutation burden (5‐7 variants) was more associated with a poor survival than the highest quartile of tumor mutation burden (>7 variants) (C, D). All multivariate analyses were stratified with age at the time of diagnosis, grade, stage, ER status, PR status, HER2 status, and radiotherapy

When patients were divided into two groups, high and low, according to the median number of detected somatic variants, high mutation burden of tumor (≥5 variants) was associated with a poor RFS (*P* = .020, HR = 2.47, 95% Cl 1.10‐6.83, Cox regression, Figure 2A) and BCSS (*P* = .009, HR = 4.35, 95% Cl 1.44‐13.16, Figure 2B). No association between the high mutation burden and OS was observed (*P* = .381). Closer analysis observed that the association of highest two quartiles of tumor mutation burden and survival were inconsistent with the hypothesis as the intermediate tumor mutation burden (5‐7 variants) was found to be more associated with a poor RFS (*P* = .001, HR = 4.35, 95% Cl 1.84‐10.26, Cox regression, Figure 2C) and BCSS (*P* = .002, HR = 6.19, 95% Cl 1.93‐19.92, Figure 2D) than the highest quartile of tumor mutation burden (>7 variants) (RFS *P* = .476, BSCC *P* = .136). The average age at the time of diagnosis was significantly (*P* = .030, unpaired samples t‐t‐test) higher in the intermediate tumor mutation group when compared to high mutation burden group. Although the age in overall was not significantly associated covariate with the RFS (*P* = .244, Cox regression) or BCSS (*P* = .143, Cox regression), the age group of ≥ 70 years old patients that was overrepresented in the intermediate mutation group was associated with the poor RFS (*P* = .050, Cox regression) and BCSS (*P* = .018, Cox regression) in statistically significant manner. No statistically significant correlation between the tumor mutation burden and BC subtypes was observed. ROC curve analysis supported the predictive ability of tumor mutation burden in predicting the relapse (AUC = 0.682, *P* = .007) while the predictive ability for BCSS and OS remained statistically non‐significant (Table S19).

### 3.5 High mutation burden of cfDNA is associated with a poor RFS

**FIGURE 3:**
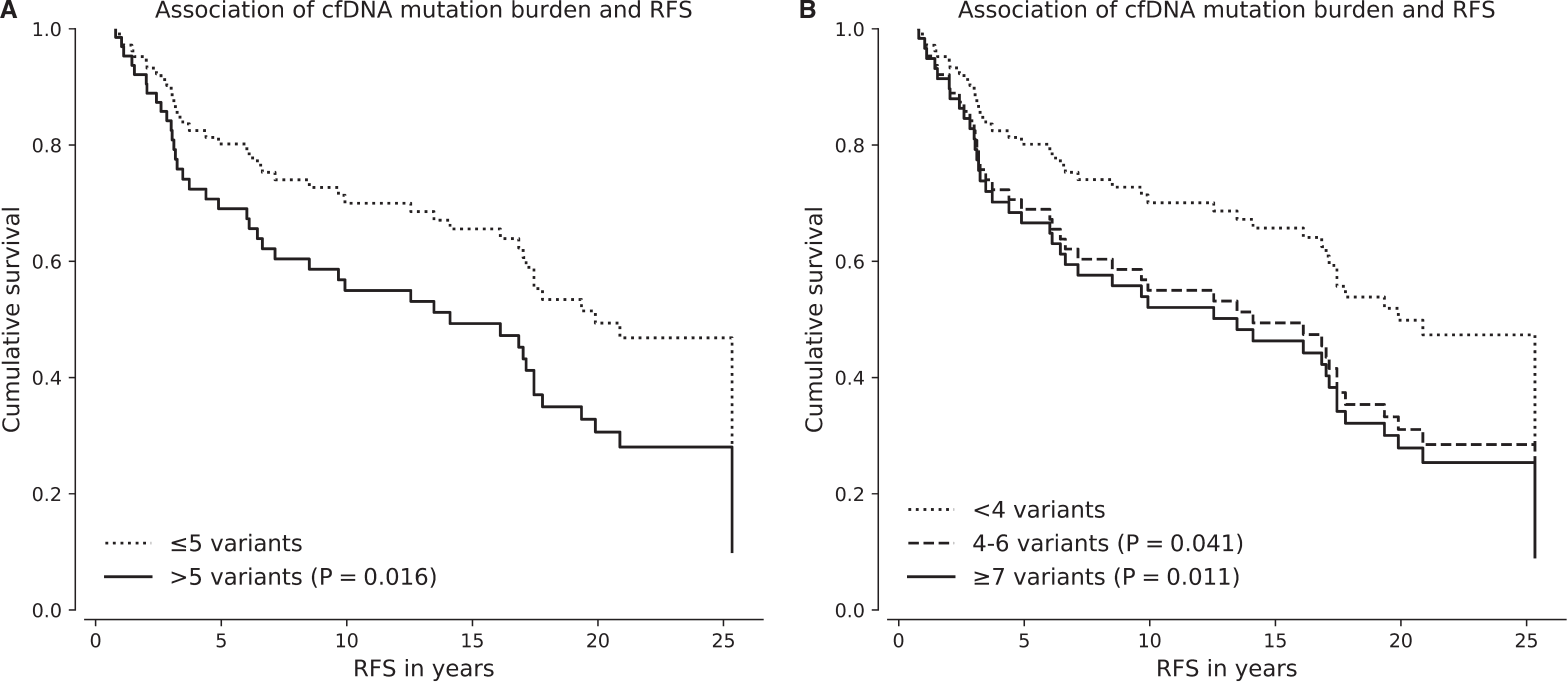
Association of cfDNA mutation burden and RFS. When patients were divided into two groups, high and low, by the median number of cfDNA mutation burden, high mutation burden of cfDNA (>5 variants) was associated with RFS (A) but not with BCSS or OS. Further analysis observed no significant difference between the highest two quartiles of cfDNA mutation burden in the terms of their association with the RFS (B). All multivariate analyses were stratified with age at the time of diagnosis, grade, stage, ER status, PR status, HER2 status, and radiotherapy

When patients were divided into two groups, high and low, according to the median number of detected somatic variants, high mutation burden of cfDNA (≥5 variants) was associated with a poor RFS (*P* = .016, HR = 2.23, 95% Cl 1.16‐4.27, Cox regression, Figure 3A). No association with the poor BCSS (*P* = .106) or OS (*P* = .473) was observed. The median split turned out to be the most effective method to classify patients as practically no difference was observed between the highest two quartiles of cfDNA mutation burden; highest quartile of cfDNA mutation burden (≥7 variants, *P* = .011, HR = 2.64, 95% Cl 1.25‐5.56, Cox regression) and intermediate cfDNA mutation burden (4‐6 variants, *P* = .041, HR = 2.27, 95% Cl 1.04‐4.99) were both associated with a poor RFS in a similar manner (Figure 3B). No statistically significant correlation between the cfDNA mutation burden and BC subtypes was observed. ROC curve analysis supported the predictive ability of cfDNA mutation burden in predicting the relapse (AUC = 0.675, *P* = .008, Table S19) while the predictive ability for BCSS and OS remained statistically nonsignificant.

### 3.6 Presence of tumor‐specific somatic variants in the cfDNA is associated with a poor RFS

**FIGURE 4:**
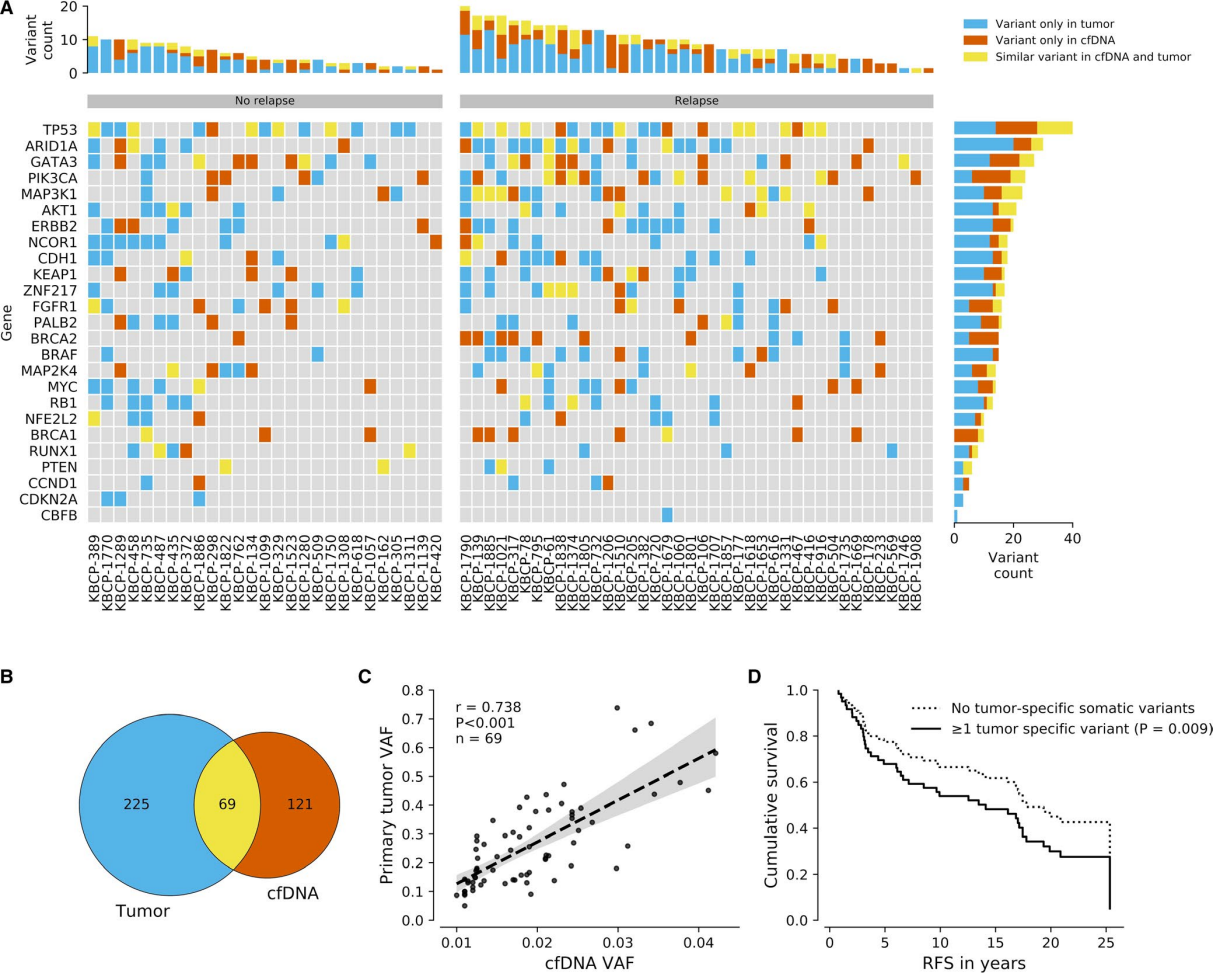
Comparison of tumor biopsy and liquid biopsy results. Somatic variants detected in the matched primary tumors and cfDNA are shown as a matrix where each column represents a single patient and each row represents a single gene (A). Bar plots describe the frequency of somatic variants per gene and per patient. Only genes sequenced in both samples were taken into account in this comparison. Venn diagram illustrates the somatic variant counts of tumor and cfDNA samples thus illustrating the discordance observed between the tumor biopsy and liquid biopsy results (B). Only genes sequenced in both samples were taken into account in this comparison. Observed discordance was mainly explained by somatic variants that were present in low VAF either in tumor or cfDNA. Indeed, a strong correlation was observed between the tumor and cfDNA VAFs of tumor‐specific somatic variants (C) suggesting that somatic variants that were presented in the tumor with a high VAF were also more likely to occur in cfDNA. Presence of tumor‐specific somatic variants was associated with a poor RFS (D) but not with the BCSS or OS. Multivariate analysis was stratified with age at the time of diagnosis, grade, stage, ER status, PR status, HER2 status, and radiotherapy

Tumor‐specific somatic variants were detected in 28 cases (45.9%). Among these cases, an average concordance between the matched tumor and cfDNA samples was 31.1 ± 0.0% when genes sequenced in both gene panels were considered. Tumor‐specific variants were most frequently detected in *TP53* (20.0% of all variants), *MAP3K1* (10.0%), *AKT1* (8.6%), *PIK3CA* (7.1%), and *GATA3* (7.1%) (Figure 4A,B). Strong correlation (*r* = 0.738, *P* < .001) was observed between the tumor and cfDNA VAFs of tumor‐specific somatic variants (Figure 4C). In general, somatic variants that were well presented in the tumor with a high VAF occurred more often also in the cfDNA. Presence of tumor‐specific variants in cfDNA was associated with a poor RFS (*P* = .009, HR = 2.31, 95% Cl 1.23‐4.31, Cox regression, Figure 4D). No association with BCSS (*P* = .201) or OS (*P* = .690) was observed. The ROC curve analysis did not support the diagnostic ability of the tumor‐specific variants in the prediction of RFS (AUC 0.521, *P* = .748, Table S19), thus suggesting that the presence of tumor‐specific somatic variants (binary variable) had more prognostic value than the number of tumor‐specific somatic variants (continuous variable).

## 4 DISCUSSION

Our results indicate that the presence of tumor‐specific somatic variants, tumor mutation burden, and cfDNA mutation burden are all associated with the poor RFS. Similar associations between the tumor‐specific somatic variants and poor RFS have been recently reported^26^, ^27^, ^28^ in the context of early stage BC patients. In contrast to these studies, our patients did not receive neoadjuvant treatment prior to sampling, thus reflecting the untreated status of cancer at the time of diagnosis.

Our results were consistent with the hypothesis and literature except for observed association between the highest two quartiles of tumor mutation burden and RFS. A closer analysis showed that the average age at the time of diagnosis was significantly higher in the intermediate tumor mutation burden group and especially the age group of ≥ 70 years old patients overrepresented in the group was associated with the poor survival. The uneven grouping of the oldest patients is probably explained by the random sampling and small cohort size. Furthermore, we cannot exclude the effect of underlying factors that were not considered in our survival analyses. Even though the reported result is unexpected, results still support the conclusion that tumor mutation burden in general is associated with the poor RFS and BCSS.

Observed discordance between the mutation profiles of the tumor tissues and matching cfDNA samples was remarkably high. It has been suggested that the discordance between the cfDNA and matched tumor in general tends to be higher in early‐stage cancers^29^, ^30^ which might explain why significantly better results in the terms of observed concordance have been obtained with advanced cancer diseases to which the majority of liquid biopsy related studies have focused. This has raised justified concerns about the accuracy of liquid‐based genotyping in the context of early‐stage cfDNA samples in clinical setup.^31^ The reasons for discordance are open for discussion and may reflect either a biological or technical variation in methodology.^32^ In this study, somatic variant calling used pooled reference sample and public databases instead of matched blood samples and thus the possibility of false somatic variant calls cannot be fully excluded despite the careful quality control for possible gDNA contaminations and false variant calls. Observed discordance relies to the assumption that the heterogeneity of disease is perfectly reflected by the tumor biopsy which is known to be questionable is some cases.^33^ As liquid biopsy is considered to reflect the systemic status of patient, we cannot exclude the possibility that some of these discordant variants may originate either from benign or metastasized tumors especially when potentially pathogenic somatic variants were detected in the serum. Observed association between the cfDNA mutation burden and RFS together with the observed discordance underlines the potential and challenges that are related to the liquid biopsy of early‐stage cancers and supports the parallel sequencing of tumor and liquid biopsies until the background of discordant variants is better understood.

It must be noted that our study has technical limitations. In addition to the variant calling without matched reference samples, used gene panels are relatively small in the context of mutation burden assessment. Although small gene panels have been used to assess mutation burden, mutation burden should be ideally evaluated from whole‐exome sequencing or whole‐genome sequencing data^34^ instead of small gene panel sequencing enriched with common oncogenes. Another issue is the age of used cohort material which is both the strength and constraint of this study. Almost thirty years long follow‐up time offers long and unique prospective perspective to the survival of Finnish BC patients who had good prognosis based on traditional prognostic parameters and allows us to detect relapses that would have been otherwise missed. At the same time, treatment strategies and techniques of BC have developed substantially. For example, neoadjuvant chemotherapy and aromatase inhibitors as an adjuvant therapy were not used when sample material was collected in the 1990s. Most liquid biopsy related studies have avoided the use of serum samples due to lysis of hematopoietic cells which may contaminate cfDNA by genomic DNA fragments.^35^ However, plasma samples were not collected in the KBCP which forced us to use serum samples in our study. Finally, it must be noted that our cohort differs from standard BC cohort material as it was specifically collected to contain patients both with and without relapsed disease.

## 5 CONCLUSIONS

To the best of our knowledge, this is the first study to report that the poor RFS of an early‐stage BC patient who have not received neoadjuvant chemotherapy can be estimated from the cfDNA sample at the time of diagnosis. We confirm the previously raised concerns about the accuracy of liquid biopsy‐based genotyping of early‐stage cancers but provide evidence that the estimate of cfDNA mutation burden and the presence of tumor‐specific somatic variants in the cfDNA may act as an independent prognostic factor and help us to identify patients with a higher risk of relapse. Further studies related to the liquid biopsy of early‐stage BC are well warranted.

## CONFLICTS OF INTEREST

The authors declare no potential conflicts of interest.

## AUTHORS’ CONTRIBUTIONS

All authors contributed to the conceptual idea of study. JK and JMH designed and performed the experiments. JK, JMH, MT, RS, and AM analyzed and interpreted the data. JK and JMH contributed equally to the writing of this manuscript. VMK and AM contributed equally to the supervision of this research project. All authors discussed the results and contributed to the final manuscript.

## Supporting information

File S1

File S2

Fig S3

Fig S4

Fig S5

Table S6‐S19

Fig S20

## Data Availability

All data are available within the manuscript. All materials are available from the corresponding author on reasonable request.
