# Supplementary figures and images for: High mutation burden of circulating cell‐free DNA in early‐stage breast cancer patients is associated with a poor relapse‐free survival
Source: Cancer Med. 2020 Jun 29;9(16):5922–31. doi: 10.1002/cam4.3258 (PMC7433819; doi:10.1002/cam4.3258)

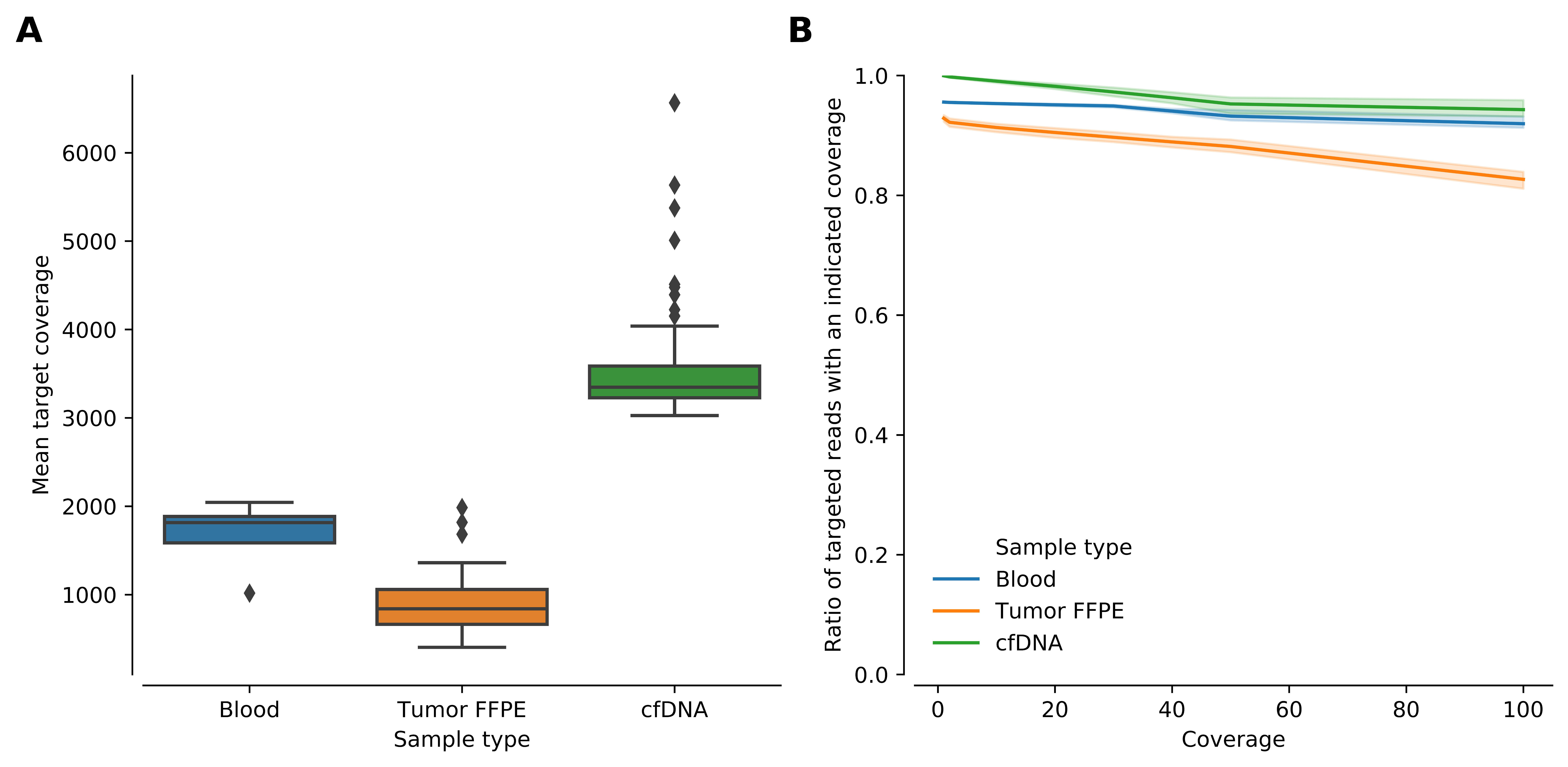

Supplement: Supplementary file 3 — Fig S3 [file CAM4-9-5922-s003.jpg]

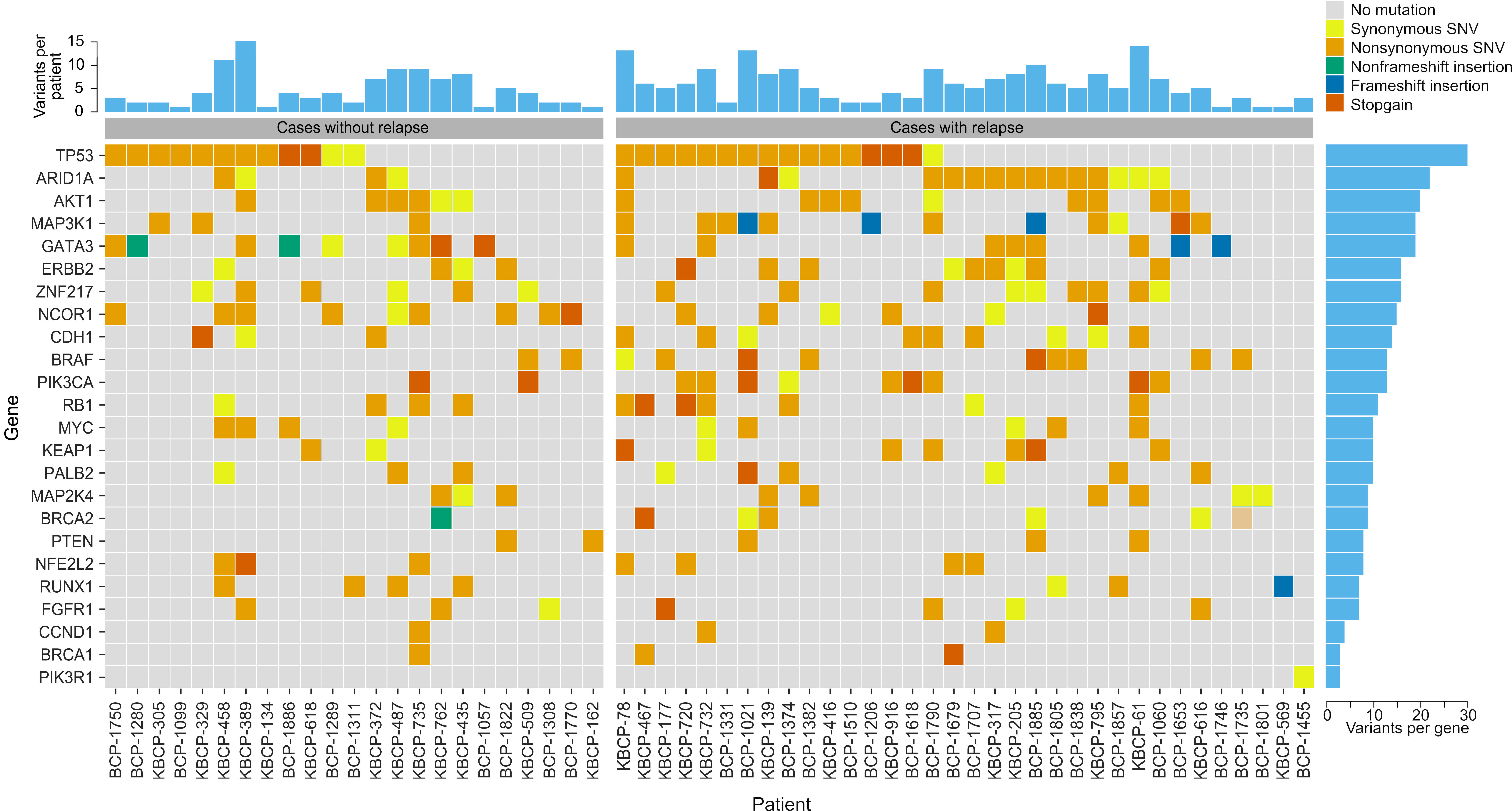

Supplement: Supplementary file 4 — Fig S4 [file CAM4-9-5922-s004.jpg]

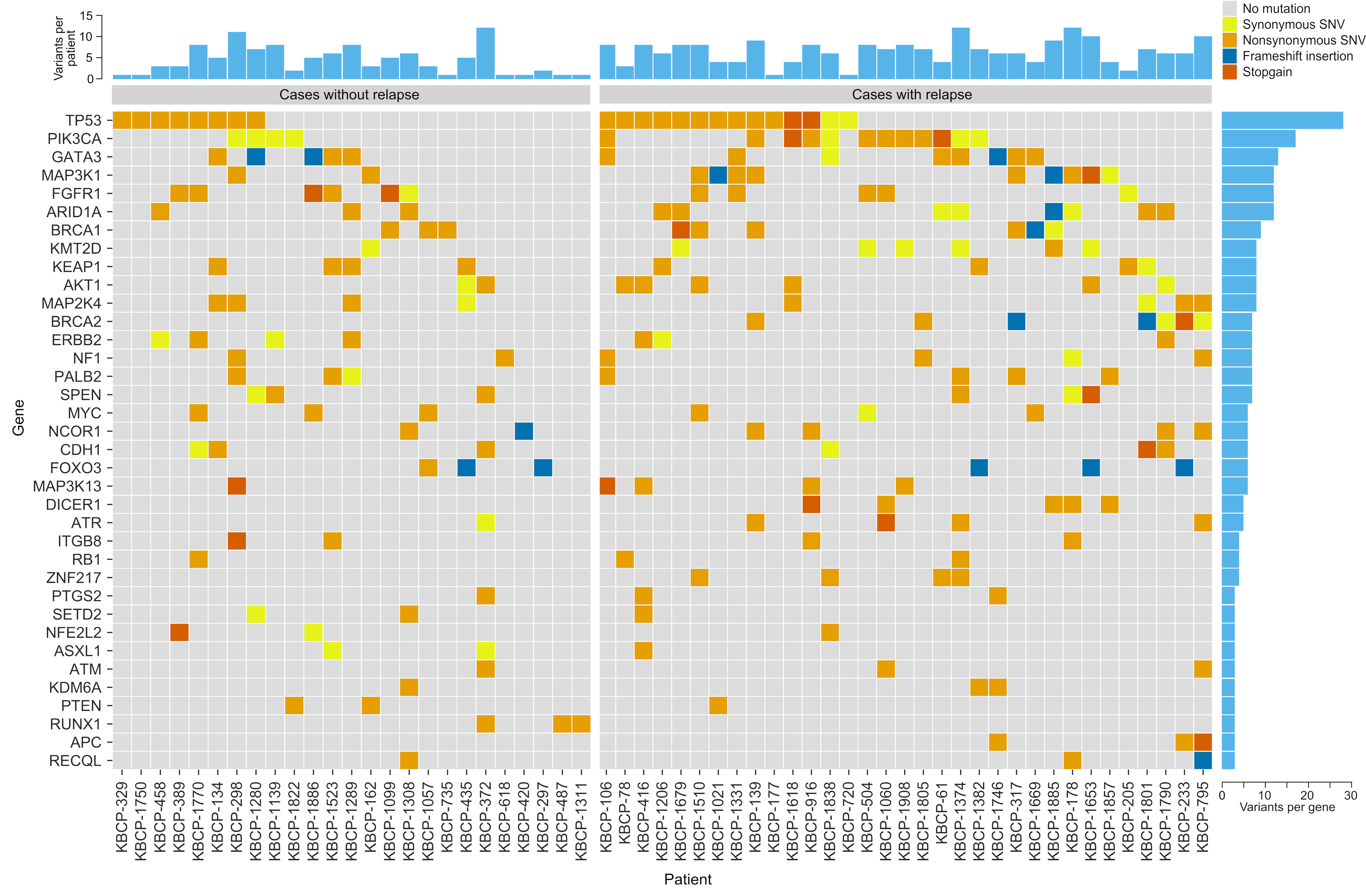

Supplement: Supplementary file 5 — Fig S5 [file CAM4-9-5922-s005.jpg]

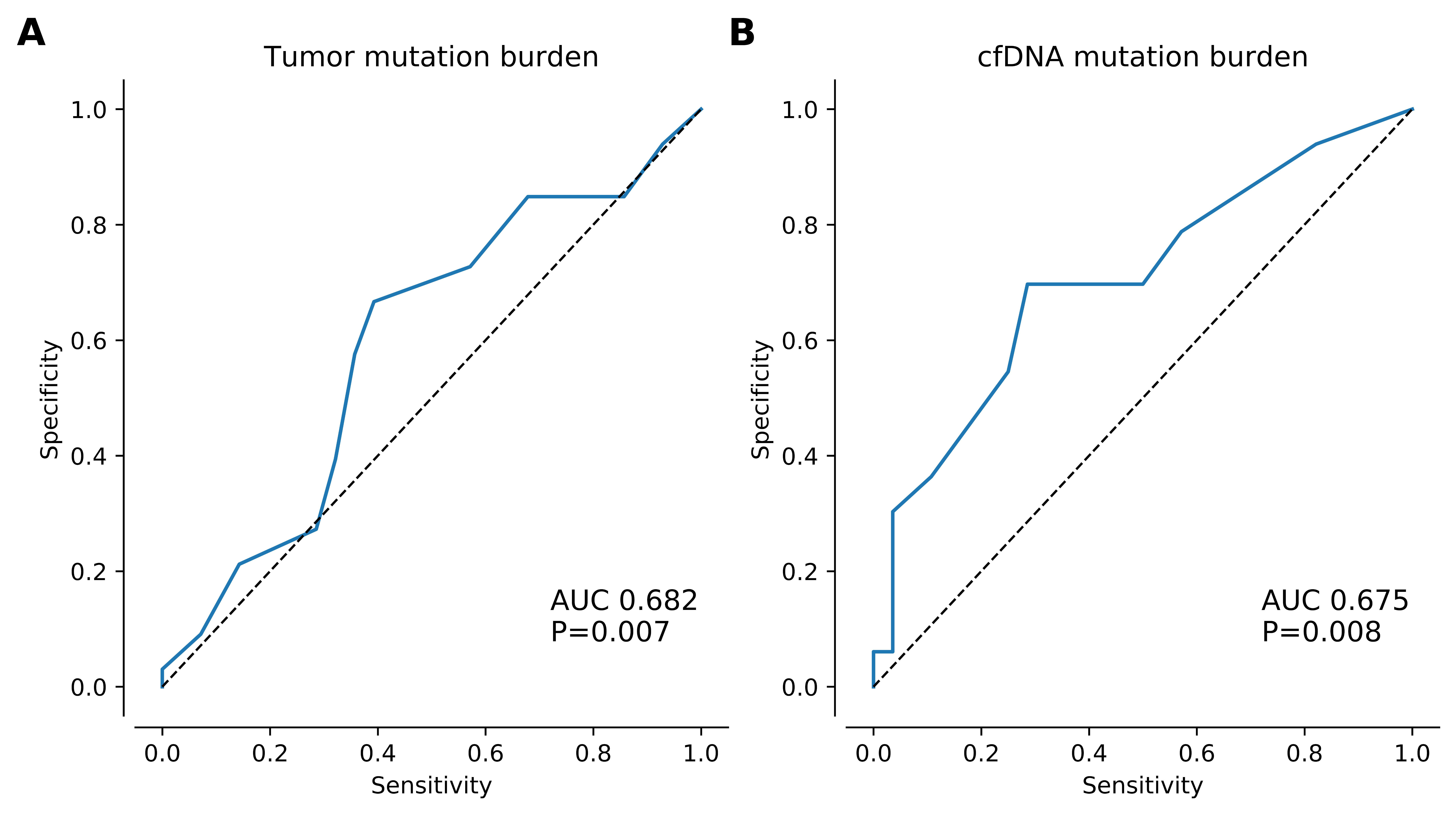

Supplement: Supplementary file 7 — Fig S20 [file CAM4-9-5922-s007.jpg]
