# Supplementary material for: High mutation burden of circulating cell‐free DNA in early‐stage breast cancer patients is associated with a poor relapse‐free survival
Source: Cancer Med. 2020 Jun 29;9(16):5922–31. doi: 10.1002/cam4.3258 (PMC7433819; doi:10.1002/cam4.3258)
Supplement: Supplementary file 6 — Table S6‐S19 [file CAM4-9-5922-s006.docx]

**Tables S6-S19**

This file contains the result tables of Cox multivariate regression analyses presented in the manuscript “High mutation burden of circulating cell-free DNA in early-stage breast cancer patients is associated with a poor relapse-free survival” by Kujala, J., Hartikainen, J.M., Tengström, M., Sironen, R., Kosma, V-M and Mannermaa, A. (2020).

Content of file:

[1. Association between tumor mutation burden and relapse-free survival 2](#_Toc38874189)

[2. Association between tumor mutation burden and breast cancer -specific survival 3](#_Toc38874190)

[3. Association between tumor mutation burden and overall survival 4](#_Toc38874191)

[4. Association between cfDNA mutation burden and relapse-free survival 5](#_Toc38874192)

[5. Association between cfDNA mutation burden and breast cancer -specific survival 6](#_Toc38874193)

[6. Association between cfDNA mutation burden and overall survival 7](#_Toc38874194)

[7. Association between tumor-specific somatic variants and relapse-free survival 8](#_Toc38874195)

[8. ROC curve statistics 9](#_Toc38874196)

# **Association between tumor mutation burden and relapse-free survival**

**Table S6.** Variables significantly associated with relapse-free survival in multivariate analysis. Age at the time of diagnosis, tumor grade, stage, ER status, PR status, HER2 status, radiotherapy and tumor mutation burden were used as covariates. Tumor mutation burden was included as two-class variable (low and high mutation burden).

| **Covariate** | **N^1^** | **B (SE)^2^** | **Wald^2^** | **p-value** | **HR (95% Cl)^4^** |
| --- | --- | --- | --- | --- | --- |
| Tumor mutation burden  Low  High | 61  28  33 | 0.903 (0.390) | Ref.^3^  5.371 | 0.020 | 2.467 (1.104 - 6.833) |
| ^1^N; Number of samples.  ^2^B(SE); Coefficient B with standard error and the Wald test value from the Cox regression survival analysis.  ^3^Ref.; Reference category  ^4^HR (95% CI); Hazard ratio of breast cancer death with 95% confidence interval from Cox regression survival analysis. | | | | | |

**Table S7.** Variables significantly associated with relapse-free survival in multivariate analysis. Age at the time of diagnosis, tumor grade, stage, ER status, PR status, HER2 status, radiotherapy and tumor mutation burden were used as covariates. Tumor mutation burden was included as three-class variable (low, intermediate, and high mutation burden).

| **Covariate** | **N^1^** | **B (SE)^2^** | **Wald^2^** | **p-value** | **HR (95% Cl)^4^** |
| --- | --- | --- | --- | --- | --- |
| Tumor mutation burden  Low  Intermediate  High | 61  28  16  17 | 1.470 (0.438)  0.351 (0.493) | 12.151  Ref.^3^  11.262  0.507 | 0.002  0.001  0.476 | 4.349 (1.843 - 10.263)  1.421 (0.540 - 3.736) |
| ^1^N; Number of samples.  ^2^B(SE); Coefficient B with standard error and the Wald test value from the Cox regression survival analysis.  ^3^Ref.; Reference category  ^4^HR (95% CI); Hazard ratio of breast cancer death with 95% confidence interval from Cox regression survival analysis. | | | | | |

# **Association between tumor mutation burden and breast cancer -specific survival**

**Table S8.** Variables significantly associated with breast cancer -specific survival in multivariate analysis. Age at the time of diagnosis, tumor grade, stage, ER status, PR status, HER2 status, radiotherapy and tumor mutation burden were used as covariates. Tumor mutation burden was included as two-class variable (low and high mutation burden).

| **Covariate** | **N^1^** | **B (SE)^2^** | **Wald^2^** | **p-value** | **HR (95% Cl)^4^** |
| --- | --- | --- | --- | --- | --- |
| Tumor mutation burden  Low  High | 61  28  33 | 1.470 (0.565) | Ref.^3^  6.771 | 0.009 | 4.349 (1.437 – 13.157) |
| ^1^N; Number of samples.  ^2^B(SE); Coefficient B with standard error and the Wald test value from the Cox regression survival analysis.  ^3^Ref.; Reference category  ^4^HR (95% CI); Hazard ratio of breast cancer death with 95% confidence interval from Cox regression survival analysis. | | | | | |

**Table S9.** Variables significantly associated with breast cancer -specific survival in multivariate analysis. Age at the time of diagnosis, tumor grade, stage, ER status, PR status, HER2 status, radiotherapy and tumor mutation burden were used as covariates. Tumor mutation burden was included as three-class variable (low, intermediate, and high mutation burden).

| **Covariate** | **N^1^** | **B (SE)^2^** | **Wald^2^** | **p-value** | **HR (95% Cl)^4^** |
| --- | --- | --- | --- | --- | --- |
| Tumor mutation burden  Low  Intermediate  High | 61  28  16  17 | 1.823 (0.596)  1.005 (0.674) | 9.639  Ref.^3^  9.358  2.221 | 0.008  0.002  0.136 | 6.192 (1.925 - 19.916)  2.731 (0.729 – 10.238) |
| ^1^N; Number of samples.  ^2^B(SE); Coefficient B with standard error and the Wald test value from the Cox regression survival analysis.  ^3^Ref.; Reference category  ^4^HR (95% CI); Hazard ratio of breast cancer death with 95% confidence interval from Cox regression survival analysis. | | | | | |

# **Association between tumor mutation burden and overall survival**

**Table S10.** Variables significantly associated with overall survival in multivariate analysis. Age at the time of diagnosis, tumor grade, stage, ER status, PR status, HER2 status, radiotherapy and tumor mutation burden were used as covariates. Tumor mutation burden was included as two-class variable (low and high mutation burden).

| **Covariate** | **N^1^** | **B (SE)^2^** | **Wald2** | **p-value** | **HR (95% Cl)^4^** |
| --- | --- | --- | --- | --- | --- |
| Age at the of diagnosis  ≤ 39 years  40-49 years  50-59 years  60-69 years  ≥ 70 years  Stage  I  II | 61  7  11  20  13  10  61  42  19 | -1.203 (0.740)  -0.552 (0.597)  -0.062 (0.614)  1.437 (0.73)  0.812 (0.361) | 20.718  Ref.^3^  2.639  0.855  0.010  4.564  Ref.^3^  5.066 | <0.001  0.104  0.355  0.920  0.033  0.024 | 0.300 (0.070 – 1.282)  0.576 (0.179 – 1.855)  0.940 (0.283 – 3.130)  4.208 (1.126 – 15.727)  2.251 (1.111 – 4.564) |
| ^1^N; Number of samples.  ^2^B(SE); Coefficient B with standard error and the Wald test value from the Cox regression survival analysis.  ^3^Ref.; Reference category  ^4^HR (95% CI); Hazard ratio of breast cancer death with 95% confidence interval from Cox regression survival analysis. | | | | | |

**Table S11.** Variables significantly associated with overall survival in multivariate analysis. Age at the time of diagnosis, tumor grade, stage, ER status, PR status, HER2 status, radiotherapy and tumor mutation burden were used as covariates. Tumor mutation burden was included as three-class variable (low, intermediate, and high mutation burden).

| **Covariate** | **N^1^** | **B (SE)^2^** | **Wald^2^** | **p-value** | **HR (95% Cl)^4^** |
| --- | --- | --- | --- | --- | --- |
| Age at the of diagnosis  ≤ 39 years  40-49 years  50-59 years  60-69 years  ≥ 70 years  Stage  I  II  Radiotherapy  No  Yes | 61  7  11  20  13  10  61  42  19  61  40  21 | -0.746 (0.791)  -0.453 (0.638)  0.420 (0.660)  2.962 (0.856)  0.923 (0.387)  1.015 (0.436) | 26.782  Ref.^3^  0.890  0.504  0.406  11.973  Ref.^3^  5.699  Ref.^3^  5.428 | <0.001  0.345  0.478  0.524  0.001  0.017  0.020 | 0.474 (0.101 – 2.234)  0.636 (0.182 – 2.219)  1.523 (0.418 – 5.553)  19.333 (3.612 – 103.485)  2.517 (1.180 – 5.369)  2.760 (1.175 – 6.483) |
| ^1^N; Number of samples.  ^2^B(SE); Coefficient B with standard error and the Wald test value from the Cox regression survival analysis.  ^3^Ref.; Reference category  ^4^HR (95% CI); Hazard ratio of breast cancer death with 95% confidence interval from Cox regression survival analysis. | | | | | |

# **Association between cfDNA mutation burden and relapse-free survival**

**Table S12.** Variables significantly associated with relapse-free survival in multivariate analysis. Age at the time of diagnosis, tumor grade, stage, ER status, PR status, HER2 status, radiotherapy and cfDNA mutation burden were used as covariates. cfDNA mutation burden was included as two-class variable (low and high mutation burden).

| **Covariate** | **N^1^** | **B (SE)^2^** | **Wald^2^** | **p-value** | **HR (95% Cl)^4^** |
| --- | --- | --- | --- | --- | --- |
| cfDNA mutation burden  Low  High | 79  40  39 | 0.800 (0.332) | Ref.^3^  5.800 | 0.016 | 2.225 (1.161 – 4.267) |
| ^1^N; Number of samples.  ^2^B(SE); Coefficient B with standard error and the Wald test value from the Cox regression survival analysis.  ^3^Ref.; Reference category  ^4^HR (95% CI); Hazard ratio of breast cancer death with 95% confidence interval from Cox regression survival analysis. | | | | | |

**Table S13.** Variables significantly associated with relapse-free survival in multivariate analysis. Age at the time of diagnosis, tumor grade, stage, ER status, PR status, HER2 status, radiotherapy and cfDNA mutation burden were used as covariates. cfDNA mutation burden was included as three-class variable (low, intermediate, and high mutation burden).

| **Covariate** | **N^1^** | **B (SE)^2^** | **Wald^2^** | **p-value** | **HR (95% Cl)^4^** |
| --- | --- | --- | --- | --- | --- |
| cfDNA mutation burden  Low  Intermediate  High | 79  40  18  21 | 0.821 (0.401)  0.971 (0.380) | Ref.^3^  4.189  6.514 | 0.041  0.011 | 2.272 (1.035 – 4.985)  2.639 (1.253 – 5.562) |
| ^1^N; Number of samples.  ^2^B(SE); Coefficient B with standard error and the Wald test value from the Cox regression survival analysis.  ^3^Ref.; Reference category  ^4^HR (95% CI); Hazard ratio of breast cancer death with 95% confidence interval from Cox regression survival analysis. | | | | | |

# **Association between cfDNA mutation burden and breast cancer -specific survival**

**Table S14.** Variables significantly associated with breast cancer -specific survival in multivariate analysis. Age at the time of diagnosis, tumor grade, stage, ER status, PR status, HER2 status, radiotherapy and cfDNA mutation burden were used as covariates. cfDNA mutation burden was included as two-class variable (low and high mutation burden).

| **Covariate** | | **N^1^** | **B (SE)^2^** | **Wald^2^** | **p-value** | **HR (95% Cl)^4^** |
| --- | --- | --- | --- | --- | --- | --- |
|  | No significantly associated covariates | | | | | |
| ^1^N; Number of samples.  ^2^B(SE); Coefficient B with standard error and the Wald test value from the Cox regression survival analysis.  ^3^Ref.; Reference category  ^4^HR (95% CI); Hazard ratio of breast cancer death with 95% confidence interval from Cox regression survival analysis. | | | | | | |

**Table S15.** Variables significantly associated with breast cancer -specific survival in multivariate analysis. Age at the time of diagnosis, tumor grade, stage, ER status, PR status, HER2 status, radiotherapy and cfDNA mutation burden were used as covariates. cfDNA mutation burden was included as three-class variable (low, intermediate, and high mutation burden).

| **Covariate** | | **N^1^** | **B (SE)^2^** | **Wald^2^** | **p-value** | **HR (95% Cl)^4^** |
| --- | --- | --- | --- | --- | --- | --- |
|  | No significantly associated covariates | | | | | |
| ^1^N; Number of samples.  ^2^B(SE); Coefficient B with standard error and the Wald test value from the Cox regression survival analysis.  ^3^Ref.; Reference category  ^4^HR (95% CI); Hazard ratio of breast cancer death with 95% confidence interval from Cox regression survival analysis. | | | | | | |

# **Association between cfDNA mutation burden and overall survival**

**Table S16.** Variables significantly associated with overall survival in multivariate analysis. Age at the time of diagnosis, tumor grade, stage, ER status, PR status, HER2 status, radiotherapy and cfDNA mutation burden were used as covariates. cfDNA mutation burden was included as two-class variable (low and high mutation burden).

| **Covariate** | **N^1^** | **B (SE)^2^** | **Wald^2^** | **p-value** | **HR (95% Cl)^4^** |
| --- | --- | --- | --- | --- | --- |
| Age at the of diagnosis  ≤ 39 years  40-49 years  50-59 years  60-69 years  ≥ 70 years  Stage  I  II | 79  7  22  24  14  12  79  60  19 | -0.782 (0.613)  -0.620 (0.583)  -0.141 (0.609)  1.510 (0.649)  0.758 (0.338) | 24.498  Ref.^3^  1.626  1.128  0.054  5.422  Ref.^3^  5.035 | <0.001  0.202  0.288  0.817  0.020  0.025 | 0.458 (0.138 – 1.522)  0.538 (0.172 – 1.689)  0.868 (0.263 – 2.866)  4.528 (1.270 – 16.141)  2.133 (1.101 – 4.135) |
| ^1^N; Number of samples.  ^2^B(SE); Coefficient B with standard error and the Wald test value from the Cox regression survival analysis.  ^3^Ref.; Reference category  ^4^HR (95% CI); Hazard ratio of breast cancer death with 95% confidence interval from Cox regression survival analysis. | | | | | |

**Table S17.** Variables significantly associated with overall survival in multivariate analysis. Age at the time of diagnosis, tumor grade, stage, ER status, PR status, HER2 status, radiotherapy and cfDNA mutation burden were used as covariates. cfDNA mutation burden was included as three-class variable (low, intermediate and high mutation burden).

| **Covariate** | **N^1^** | **B (SE)^2^** | **Wald^2^** | **p-value** | **HR (95% Cl)^4^** |
| --- | --- | --- | --- | --- | --- |
| Age at the of diagnosis  ≤ 39 years  40-49 years  50-59 years  60-69 years  ≥ 70 years  Stage  I  II | 79  7  22  24  14  12  79  60  19 | -0.782 (0.613)  -0.620 (0.583)  -0.141 (0.609)  1.510 (0.649)  0.758 (0.338) | 24.498  Ref.^3^  1.626  1.128  0.054  5.422  Ref.^3^  5.035 | <0.001  0.202  0.288  0.817  0.020  0.025 | 0.458 (0.138 – 1.522)  0.538 (0.172 – 1.689)  0.868 (0.263 – 2.866)  4.528 (1.270 – 16.141)  2.133 (1.101 – 4.135) |
| ^1^N; Number of samples.  ^2^B(SE); Coefficient B with standard error and the Wald test value from the Cox regression survival analysis.  ^3^Ref.; Reference category  ^4^HR (95% CI); Hazard ratio of breast cancer death with 95% confidence interval from Cox regression survival analysis. | | | | | |

# **Association between tumor-specific somatic variants and relapse-free survival**

**Table S18.** Variables significantly associated with overall survival in multivariate analysis. Age at the time of diagnosis, tumor grade, stage, ER status, PR status, HER2 status, radiotherapy and presence of tumor-specific somatic variants in the cfDNA were used as covariates. Presence of tumor-specific somatic variants in the cfDNA was included as two-class variable.

| **Covariate** | **N^1^** | **B (SE)^2^** | **Wald^2^** | **p-value** | **HR (95% Cl)^4^** |
| --- | --- | --- | --- | --- | --- |
| Presence of tumor-specific somatic variants in the cfDNA  No  Yes | 61  33  28 | 0.835 (0.319) | Ref.^3^  24.498 | 0.009 | 2.305 (1.234 – 4.306) |
| ^1^N; Number of samples.  ^2^B(SE); Coefficient B with standard error and the Wald test value from the Cox regression survival analysis.  ^3^Ref.; Reference category  ^4^HR (95% CI); Hazard ratio of breast cancer death with 95% confidence interval from Cox regression survival analysis. | | | | | |

# **ROC curve statistics**

**Table S19.** ROC curve statistics for tumor and cfDNA mutation burden and presence of tumor-specific somatic variants.

| **Covariate** | **Predicted event** | **AUC (95% Cl)^1^** | **p-value** |
| --- | --- | --- | --- |
| Tumor mutation burden  cfDNA mutation burden  Presence of tumor-specific somatic variants | RFS  BCSS  OS  RFS  BCSS  OS  RFS  BCSS  OS | 0.682 (0.356 – 0.621)  0.609 (0.470 – 0.748)  0.488 (0.356 – 0.621)  0.675 (0.556 – 0.794)  0.557 (0.419 – 0.696)  0.509 (0.376 – 0.641)  0.521 (0.393 – 0.650)  0.507 (0.368 – 0.646)  0.507 (0.375 – 0.639) | 0.007  0.121  0.864  0.008  0.414  0.900  0.748  0.924  0.916 |
| ^1^AUC (95% Cl); Area under curve with 95% confidence interval | | | |
